# Subcutaneous Tumor Tract Seeding After Percutaneous Ablation for Clear Cell Renal Cell Carcinoma: A Case Report and Literature Review

**DOI:** 10.3390/diagnostics16020231

**Published:** 2026-01-11

**Authors:** Agostino Fraia, Filippo Caudana, Francesco Di Bello, Sara Riolo, Salvatore Papi, Dario Brunello, Ivan Di Giulio, Giovanni Costa, Roberto Knez, Tommaso Silvestri, Bernardino de Concilio, Riccardo Bertolo, Massimiliano Creta, Alessandro Antonelli, Nicola Longo, Guglielmo Zeccolini, Antonio Celia

**Affiliations:** 1 Department of Neurosciences, Reproductive Sciences and Odontostomatology, University of Naples “Federico II”, 80131 Naples, Italy; fran.dibello12@gmail.com (F.D.B.); salvatorepapi@blu.it (S.P.); max.creta@gmail.com (M.C.); nicola.longo@unina.it (N.L.); 2 Urology Unit, Department of Surgery, Dentistry, Pediatrics and Gynecology, Azienda Ospedaliera Universitaria Integrata, University of Verona, 37126 Verona, Italy; caudana.filippo997@gmail.com (F.C.); dario.brunello@studenti.univr.it (D.B.);; 3 Department of Urology, La Sapienza University, 00185 Rome, Italy; sara.riolo@uniroma1.it; 4 Department of Urology, San Bassiano Hospital, 36061 Bassano del Grappa, Italy; ivan.digiulio@aulss7.veneto.it (I.D.G.); giovanni.costa1@aulss7.veneto.it (G.C.); roberto.knez@aulss7.veneto.it (R.K.); tommaso.silvestri@aulss7.veneto.it (T.S.); bernardinodeconcilio@hotmail.com (B.d.C.); guglielmo.zeccolini@aulss7.veneto.it (G.Z.); antonio.celia@aulss7.veneto.it (A.C.)

**Keywords:** renal cell carcinoma, percutaneous ablation, cryoablation, microwave, radiofrequency, tumor seeding, tumor tract seeding, complication, rare

## Abstract

**Background and Clinical Significance**: Percutaneous ablation is an increasingly used nephron-sparing treatment for small renal masses (SRMs). Although generally considered safe, tumor seeding along the applicator tract is rare (<0.1%) and may be underreported. This study reviews the existing literature to synthesize patterns, potential risk factors, and clinical presentation of this complication following percutaneous thermal ablation of renal cell carcinoma (RCC). **Case Presentation:** We report the case of an 84-year-old man who developed late subcutaneous abdominal-wall tumor seeding more than ten years after nephron-sparing surgery for a T1a renal mass and following three sessions of percutaneous cryo- and microwave ablation for recurrent clear-cell renal cell carcinoma (ccRCC). The lesion was surgically excised, and histology confirmed ccRCC with negative margins. A descriptive literature review was conducted using PubMed and ScienceDirect to identify English-language case reports and case series (CS) documenting tumor seeding after RCC percutaneous ablation. Eight studies involving nine patients met the inclusion criteria. The median age was 66 years (interquartile range [IQR] 64–74; range 47–84). The median follow-up duration was 11 months (IQR, 4.5–18.5; range 3–60), and the median interval to tumor seeding was 11 months (IQR, 6–18.5; range 3–60). Management included surgical excision (50%), repeat cryoablation (25%), and systemic therapy or supportive care (25%). **Conclusions**: Tumor tract seeding following percutaneous ablation for RCC is rare, with variable latency and presentation. Procedural factors such as the absence of tract ablation, multiple probe passes, and intra-procedural biopsy may increase risk. Awareness of this complication and long-term surveillance should be incorporated into follow-up protocols. Despite this risk, percutaneous ablation remains a safe and effective option for appropriately selected patients.

## 1. Introduction

RCC is the third most common urological malignancy and the predominant histopathological subtype of renal tumors [1]. The widespread use of cross-sectional imaging has increased the incidental detection of SRMs, with up to 87% of newly diagnosed renal cell carcinomas classified as stage T1a lesions [2]. While partial nephrectomy remains the gold standard for localized disease, percutaneous thermal ablation techniques, including cryoablation (CA), microwave ablation (MWA), and radiofrequency ablation (RFA), have gained popularity as nephron-sparing and minimally invasive alternatives for patients who are not surgical candidates or who prefer less invasive options. These modalities provide excellent local control and preserve renal function, with intermediate-term oncologic outcomes comparable to partial nephrectomy in carefully selected patients at experienced centers [3,4]. Nevertheless, rare complications may arise, such as tumor seeding along the needle tract. Tumor seeding refers to the implantation and subsequent growth of viable tumor cells along the probe tract, potentially presenting as subcutaneous or retroperitoneal nodules adjacent to the ablation site [5]. Historically, this complication has been considered anecdotal (<0.01%). However, the true incidence remains uncertain, as most available evidence consists of case reports and small case series, and detection may depend on the duration and rigor of imaging follow-up or the availability of histological confirmation [6,7]. Although techniques such as coaxial systems and tract cauterization are thought to minimize this risk, reported cases demonstrate that tumor seeding can still occur despite these precautions [4,7].

In this review, we present a case of histologically confirmed tumor seeding following percutaneous renal CA. The recurrence was detected several years after the initial treatment. All three recurrences were managed with percutaneous ablation. Each lesion was localized and technically suitable for a percutaneous approach, which was preferred due to the patient’s comorbidities and personal preference. We also reviewed the existing literature, summarizing clinical characteristics, potential risk factors, and outcomes. This dual approach aims to increase awareness of this rare complication and highlight the importance of meticulous procedural technique and consistent long-term follow-up in patients undergoing percutaneous ablation for RCC.

## 2. Case Report

**Figure 1:**
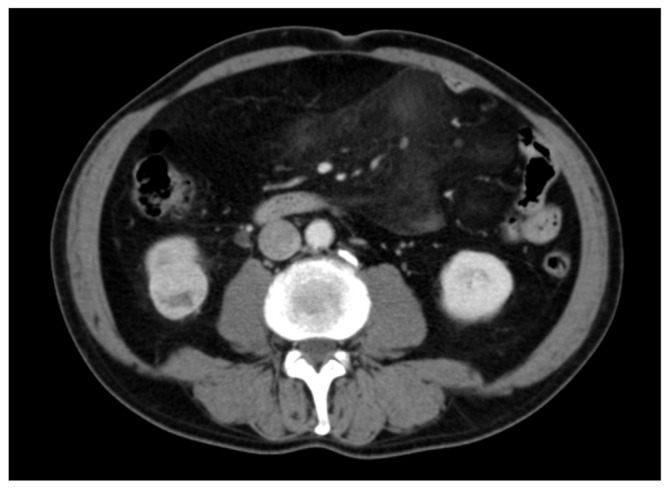
Axial CT image demonstrating a 3.5 cm contrast-enhancing mass in the right lower pole of the kidney.

**Figure 2:**
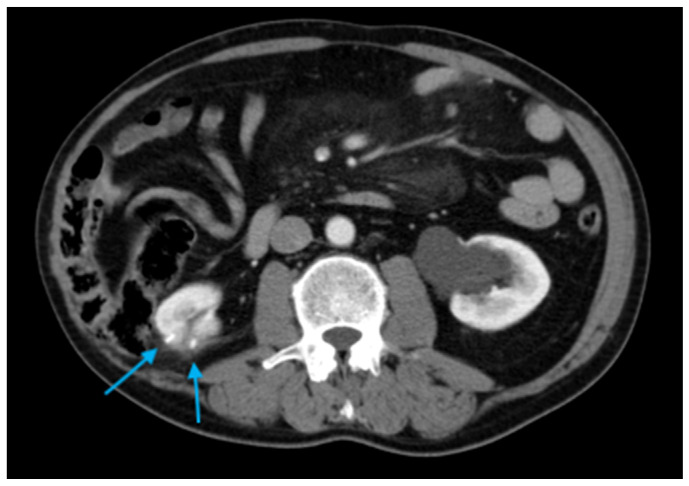
Axial CT surveillance image at 6 months demonstrating the prior tumor resection bed, with no evidence of local recurrence, and hem-o-lok clips (*light blue arrows*).

An 84-year-old Italian man presented to our department with a 3.5 cm contrast-enhancing mass in the lower pole of the right kidney, discovered incidentally during a computed tomography (CT) scan conducted for unrelated reasons (Figure 1). His medical history included hypertension, hyperlipidemia, and stable coronary artery disease. Laboratory results were unremarkable. In September 2003, the patient underwent a right partial laparoscopic tumor enucleation without any perioperative or postoperative complications. Histopathological examination revealed a ccRCC with Fuhrman grade I and negative surgical margins. Postoperative surveillance CT scans remained unremarkable for 12 years (Figure 2).

**Figure 3:**
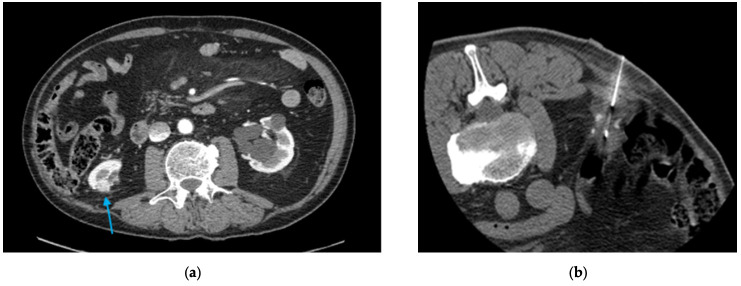
(**a**) Axial follow-up CT image depicting a 1.8 cm contrast-enhancing nodule indicating local recurrence (*light blue arrow*); (**b**) Axial CT image during renal CA showing the probe traversing the right flank. The patient was positioned prone during the procedure.

In May 2015, a contrast-enhanced CT scan performed for left-sided abdominal pain revealed a 1.8 cm contrast-enhancing nodule adjacent to the previous tumor resection bed. A CT-guided percutaneous renal biopsy using an 18-gauge core biopsy system confirmed recurrent ccRCC. The lesion was treated during the same session with percutaneous CA (Figure 3). Immediate and three-month follow-up imaging demonstrated satisfactory ice-ball coverage with no residual contrast enhancement.

**Figure 4:**
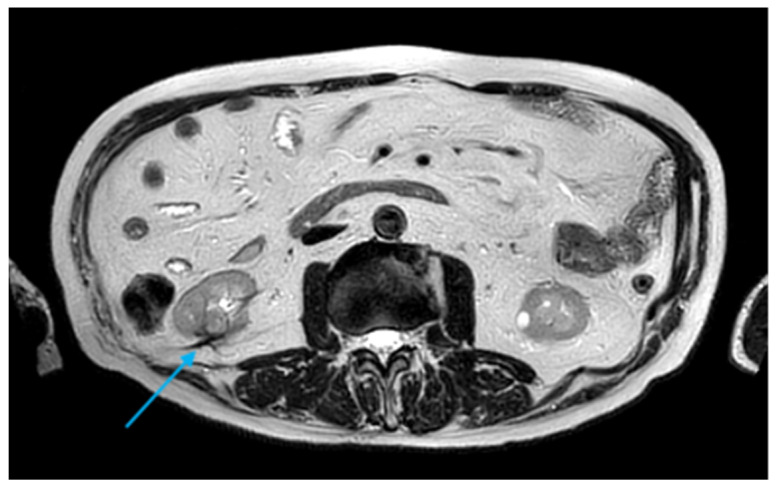
Axial T2-weighted MRI image demonstrating a 1.6 cm nodule indicative of local recurrence (*light blue arrow*).

In May 2018, a suspicious second recurrence measuring 1.6 cm was detected on magnetic resonance imaging (MRI) and was managed with repeat CA following a confirmatory biopsy (Figure 4). Contrast-enhanced ultrasound (CEUS) at 3 and 8 months demonstrated complete devascularization. All CA procedures were performed using the Visual-ICE^®^ System (Galil Medical Inc., Saint Paul, MN, USA). Different types of CryoNeedle™ (Galil Medical Inc., Saint Paul, MN, USA) were selected based on lesion anatomy and morphology. The probes used included the IceRod^®^ 1.5 mm and the IceSphere^®^ 1.5 mm. The patient was positioned prone or oblique in the CT scanner under conscious sedation, and a tumor biopsy was performed using an 18-gauge core needle prior to ablation. After probe placement, CA was carried out using a double freeze–thaw cycle: a 10 min freezing cycle at temperatures below −40 °C, followed by a 10 min passive thawing cycle. During the freezing phase, repeated control ablations with sequential CT acquisitions were conducted to monitor ice-ball expansion and prevent injury to surrounding organs. At the end of the procedure, active helium thawing facilitated the rapid removal of cryoprobes, followed by a final CT scan to detect major complications. Primary technical success was defined as competition of a full freeze–thaw–freeze cycle, with the ice ball extending at least 5 mm beyond the tumor margins.

**Figure 5:**
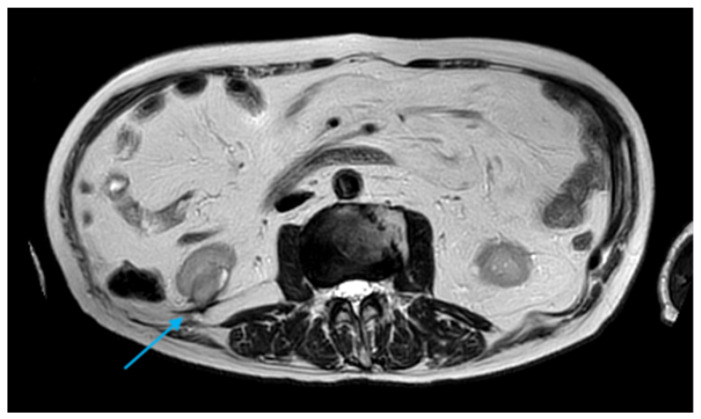
Axial T2-weighted MRI image demonstrating a 2.5 × 2 cm nodule indicative of local recurrence (*light blue arrow*).

In October 2019, follow-up CEUS raised suspicion of a third recurrence, measuring 2 cm at the inferior pole of the right kidney. Subsequent MRI confirmed an enhancing lesion, measuring 2.5 × 2 cm, which was treated with percutaneous MWA (Figure 5).

**Figure 6:**
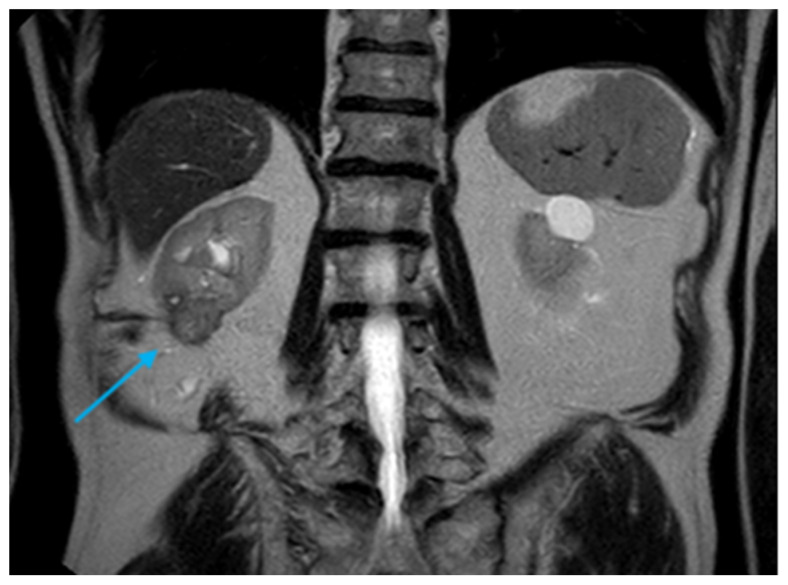
Coronal T2-weighted MRI image demonstrating nodular growth observed during follow-up (*light blue arrow*).

In November 2020, follow-up MRI showed an increase in size of the treated renal lesion to 2.5 cm, as well as the appearance of an additional 11 mm contrast-enhancing nodule in the right posterolateral abdominal wall, located just beneath the percutaneous probe entry site (Figure 6).

**Figure 7:**
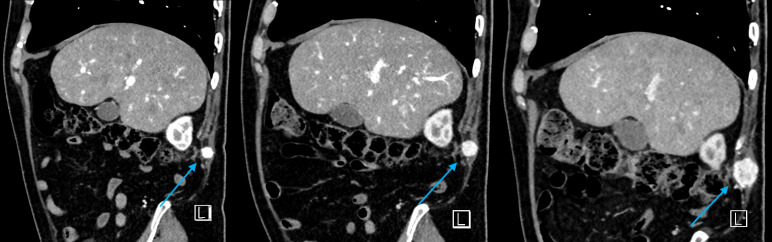
Sagittal CT images demonstrating dimensional progressive enlargement of the subcutaneous nodule during follow-up, measuring 1.31 cm, 1.55 cm, and 1.80 cm, respectively (*light blue arrows*).

In January, the patient was presented with two treatment options: right laparoscopic nephrectomy or repeat CA of the renal lesion with surveillance of the abdominal wall nodule. After a shared decision-making process, CA was chosen due to the patient’s cardiac comorbidities, and CA was performed in March 2021. Subsequent contrast-enhanced CT scans performed over the following years showed no recurrence of the right renal tumor. However, progressive enlargement of the abdominal wall nodule was observed (Figure 7).

**Figure 8:**
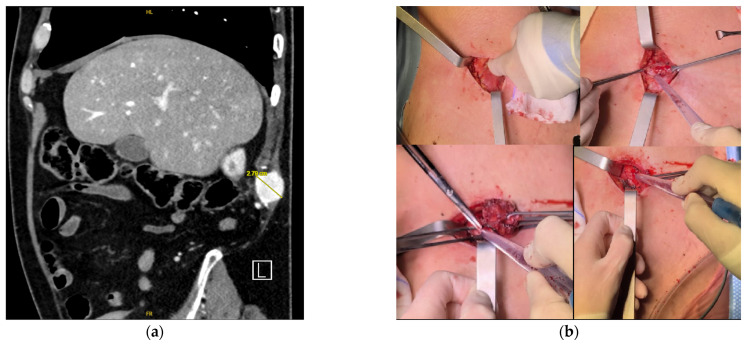
(**a**) Sagittal CT image showing a nodule measuring 2.79 cm; (**b**) Following the skin incision, retractors are used to hold the wound edges apart as the surgeon palpates the subcutaneous cavity to locate the lesion and evaluate tissue planes. Two retractors are positioned to create the surgical field. Using forceps and suction, the surgeons carefully separate the tissue layers to achieve clear access to the target area. The central fragment is grasped with forceps, while suction maintains a clear, blood-free field, allowing precise excision of the lesion. Before closure, hemostasis is confirmed by aspirating any residual blood and thoroughly inspecting the margins of the surgical cavity.

**Figure 9:**
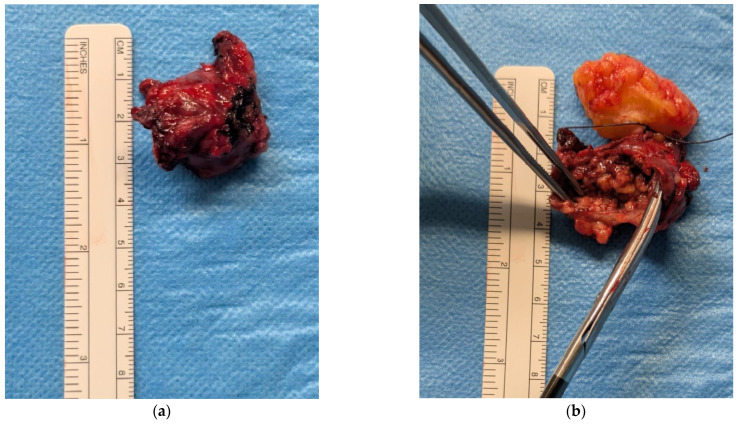
(**a**) Macroscopic view of the freshly excised specimen: approximately 3 cm tissue nodule placed beside a ruler for measurement. (**b**) Gentle spreading with forceps reveals internal adipose tissue and a central area of necrosis, with a sutured fragment of subcutaneous fat for anatomical orientation.

As a result, surgical excision was performed in June 2025 through an elliptical skin incision (Figure 8). Histological examination revealed a subcutaneous clear-cell carcinoma, consistent with metastasis from the primary renal tumor. Resection margins were negative (Figure 9). The postoperative course was uneventful.

## 3. Materials and Methods

A descriptive literature review was conducted to evaluate reported cases of tumor seeding following thermal percutaneous ablation for RCC. The PRISMA 2020 flow diagram illustrates the search and selection process; no systematic review was conducted.

### 3.1. Statistical Analysis

Only descriptive statistics were used. Continuous variables were reported as medians and IQR, or as counts with percentages for categorically coded variables. In all statistical analyses, Microsoft Excel (Microsoft Corporation, Redmond, WA, USA) was used for statistical computing.

### 3.2. Literature Search

The PubMed (U.S. National Library of Medicine, Bethesda, MD, USA) and ScienceDirect (Elsevier, Amsterdam, The Netherlands) databases were searched through 29 June 2025. The search strategy included combinations of the following keywords, filtered for case reports: renal cell carcinoma AND percutaneous ablation AND tumor seeding; renal cell carcinoma AND cryoablation AND tumor seeding; renal cell carcinoma AND microwave ablation AND tumor seeding; renal cell carcinoma AND radiofrequency ablation AND tumor seeding; renal cancer AND seeding AND cryoablation AND retroperitoneoscopic; renal cell carcinoma AND tumor tract seeding AND needle manipulation; neoplastic seeding AND cryoablative AND renal masses AND case series. Reference lists of relevant publications were also screened for additional eligible studies.

### 3.3. Selection Criteria

Two independent reviewers (A.F. and F.C.) screened the titles, abstracts, and full texts of all retrieved articles to determine eligibility. Disagreements were resolved through discussion and, if necessary, consultation with a third reviewer (T.S.). Studies were included if they were written in English, available in full text, and described one or more cases of histologically confirmed tumor tract seeding following thermal percutaneous ablation for RCC. Articles lacking sufficient procedural or clinical detail were excluded.

### 3.4. Data Collection

For each eligible study, data were extracted on patient demographics, tumor histology and grade, ablation modality, needle and probe characteristics, and whether tract ablation was performed. Data on clinical presentation, timing, and site of seeding, treatment, follow-up duration, and oncologic outcomes were also collected. Data extraction was carried out independently by both reviewers and cross-checked for consistency and accuracy. Findings were summarized descriptively.

In addition to the literature review, we reported a case of subcutaneous tumor-tract seeding following multiple percutaneous ablations for recurrent RCC. Data collection was performed in accordance with ethical standards, and written informed consent was obtained from the patient for the publication of clinical information and images.

## 4. Results

**Figure 10:**
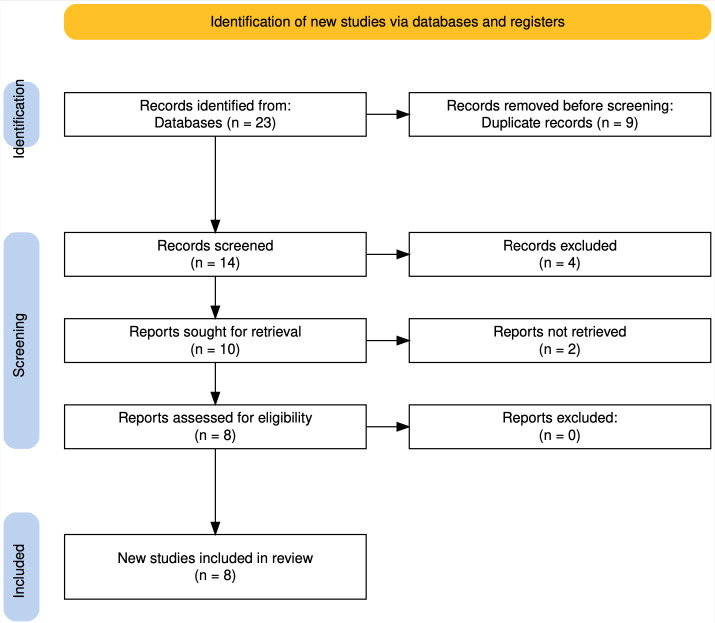
PRISMA 2020 flow diagram depicting inclusion and exclusion criteria of tumor seeding following thermal percutaneous ablation for RCC [8].

The systematic search identified 23 studies. After screening titles and abstracts, 14 papers were considered eligible for inclusion. Following full-text assessment for relevance and methodological rigor, six studies were excluded. Ultimately, eight studies addressing tumor seeding after percutaneous ablation, including seven case reports and one CS, met the inclusion criteria and were analyzed in detail (Figure 10). Across these studies, 11 patients were initially reported; however, two were excluded due to duplicate data and lack of ablative treatment, respectively.

### 4.1. Patient Demographics

Study characteristics and patient demographics are summarized in Table 1. The median age at diagnosis was 66 years (IQR 64–74; range 47–84), with a mean of 67.6 ± 10.1 years. Three patients (33.3%) were female, and five (55.6%) were male.

### 4.2. Tumor and Needle Manipulation Characteristics

Tumor and needle manipulation characteristics are summarized in Table 2. Initial diagnostic procedures included nephron-sparing surgery (NSS) in four cases (44.4%) and percutaneous or surgical biopsy in five cases (55.6%). Among patients who underwent NSS, three procedures (75%) were performed via an open approach, and the surgical approach was not reported in one case (25%). Of the biopsy-based diagnoses, two procedures (40%) were CT-guided, one (20%) was performed using a laparoscopic retroperitoneal approach, and two studies (40%) did not specify the imaging modality used. Histopathological evaluation revealed ccRCC in five cases (55.6%), papillary renal cell carcinoma (pRCC) in two cases (22.2%), and unspecified RCC in two cases (22.2%). Fuhrman grading was reported in five cases: two were Grade 1 (22.2%), one was Grade 2 (11.1%), one was Grade 3 (11.1%), and one ranged from Grade 2 to 4 (11.1%). Three cases (33.3%) did not report Fuhrman grade, and one case (11.1%) indicated that it was not specified in the histopathological examination.

CA was the predominant treatment modality, performed in eight cases (88.9%), while RFA was used in one case (11.1%). Multiple percutaneous ablations were performed in four cases (44.4%): RFA in one case (11.1%) and CA in three cases (33.3%). Needle gauge was specified in four cases (44.4%): 16-gauge in one case (11.1%), 20-gauge in two cases (22.2%), and both 19-gauge and 20-gauge in one case (11.1%). The remaining five studies (55.6%) did not report the needle gauge. Details regarding ablation probes were available in five studies (55.6%), while four (44.4%) did not specify the number or type of probes used. Coaxial systems were employed in two cases (22.2%). Needle tract ablation was documented in only one case (11.1%); the remaining eight studies (88.9%) either did not perform this procedure or failed to report it.

### 4.3. Follow-Up and Outcomes of Tumor Seeding

Follow-up and outcome data are summarized in Table 3. Follow-up data were available for eight cases (88.9%). Post-ablation surveillance imaging consisted of CT scan in six cases (66.7%), MRI in one case (11.1%), and a combination of CEUS with either MRI or CT scan in two cases (22.2%). The median follow-up duration was 11 months (IQR 4.5–18.5; range 3–60), with a mean of 16.4 ± 18.7 months. The median interval to tumor seeding was 11 months (IQR 6–18.5 months; range 3–60 months) with a mean of 16.8 ± 18.5 months. All tumor seeding events were detected radiologically: seven cases (77.8%) presented as multiple nodules and two (22.2%) as single lesions. Tumor seeding sites were reported in all cases and included varied anatomical regions: two cases (22.2%) involved the intraperitoneal space, two (22.2%) the perinephric fat, one (11.1%) the perinephric fat and lumbar musculature, two (22.2%) the lumbar region, one (11.1%) the cryoprobe tract, and one (11.1%) the retroperitoneum.

Histopathological confirmation of tumor seeding was available in seven cases (77.8%). Of these, five lesions (55.6%) were ccRCC, two (22.2%) were pRCC, one specimen (11.1%) was unspecified RCC, and one (11.1%) was non-assessable. Management strategies were reported in eight cases (88.9%). Four patients (50.0%) underwent surgical intervention, including two local excisions and two radical nephrectomies. Two patients (25.0%) underwent repeat CA, one (12.5%) received systemic chemotherapy, and one (12.5%) was managed with supportive care alone.

Clinical outcomes were available for six patients (66.7%). Three patients (33.3%) remained disease-free following local therapy, while three (33.3%) experienced disease progression, including two who developed metastatic disease.

## 5. Discussion

Needle tract tumor seeding following ablative therapies for RCC is a rare yet critical complication that may have significant clinical implications. This review provides the most comprehensive synthesis to date of reported cases of tumor seeding after percutaneous thermal ablation for RCC. By consolidating nine published cases, we aimed to describe the oncologic patterns, procedural risk factors, and clinical outcomes associated with this rare event. As percutaneous ablation techniques such as CA and RFA are increasingly adopted, a clear understanding of potential risks is essential for patient counseling, procedural planning, and surveillance [7].

The reviewed cohort consisted predominantly of older adults, with a median age of 66 years and a slight male predominance (55.6%), a demographic profile somewhat older than typically observed in salvage RCC studies [16]. Although the gender distribution was relatively balanced, incomplete demographic reporting highlights the need for standardized data collection. Histologically, ccRCC was the most common subtype (55.6%), reflecting global epidemiologic trends [1]. However, incomplete Fuhrman grade reporting limited assessment of tumors aggressiveness.

From a procedural standpoint, CA was the primary modality (88.9%), consistent with its widespread use and favorable safety profile. However, key technical details, such as needle gauge, probe type, use of coaxial systems, and, particularly, tract cauterization, were reported inconsistently. Seven cases lacked documentation regarding the use of coaxial sheaths, preventing firm conclusions about modifiable risk factors. It is unclear whether this reflects underutilization or underreporting [4,7,11]. Previous studies, including those by Akravein et al. and Krambeck et al., recommend techniques such as “hot withdrawal,” tract cauterization, and coaxial systems to reduce cell displacement during probe manipulation [7,9]. Although tract ablation is advocated to minimize tumor cell dissemination, it was performed in only one case (11.1%). More consistent application of tract ablation may help reduce risk, but supporting evidence is limited. Notably, tumor seeding occurred even in cases using coaxial systems, indicating that these precautions are not entirely fail-safe. As emphasized by Krambeck et al. and van de Kamp et al., minimizing probe repositioning and avoiding intraoperative biopsy of high-risk lesions may further reduce risk [9,10].

Institutional data from our center demonstrated a tumor seeding rate of approximately 0.45%, corresponding to one case among 224 ablation procedures (176 percutaneous CA, 24 laparoscopic CA, and 24 MWA). All patients underwent standardized imaging follow-up with CT scan or MRI performed at 6, 12, 18, 24, 36, 48, and 60 months post-treatment. Our tumor seeding rate is slightly lower than the 0.7% reported by Viswanathan et al., but higher than historical estimates of less than 0.01% [11,13]. This discrepancy likely reflects underrecognition, shorter follow-up periods, or variability in reporting practices. Shen et al. noted that many CA series lack standardized long-term surveillance and use varying definitions of treatment success, complicating incidence calculations [13].

In our review, the median follow-up duration was 11 months (IQR 4.5–18.5; range 3–60 months), with a mean follow-up of 16.4 ± 18.7 months, highlighting the variability in surveillance periods and the potential for delayed presentations. The median time to seeding was 11 months (IQR 6–18.5; range 3–60 months) with a mean of 16.8 ± 18.5 months, emphasizing the importance of extending surveillance beyond conventional post-ablation intervals. The diverse seeding locations, including the fat, retroperitoneum, lumbar musculature, intraperitoneal spaces, and the cryoprobe tract, support the possibility of multiple dissemination mechanisms. Atypical sites, such as the rectovesical pouch, suggest that intratumoral pressure changes, mechanical trauma, or hematogenous spread may contribute to seeding [10,12]. Van de Kamp et al. also proposed that intraoperative biopsy, cryoprobe manipulation, and CO_2_ insufflation may facilitate tumor cells dispersion during laparoscopic procedures [7,10,15].

Management strategies in the reported cases were heterogeneous, including repeat ablation, surgical excision, radical nephrectomy, systemic therapy, and supportive care. Despite this variability, outcomes were not uniformly poor: one-third of patients achieved disease-free status following local treatment, while another third experienced disease progression, including two who developed metastases. As reported by Viswanathan et al. and Rizzo et al., indolent tumors often remain localized after seeding, whereas high-grade lesions exhibit early systemic progression [11,12]. Overall, this review highlights the technical and oncologic challenges of managing RCC recurrence following ablative therapy. While percutaneous ablation remains a valuable option, particularly for patients with significant comorbidities or limited renal reserve, clinicians must weigh its benefits against the potential risk of tumor dissemination. Vigilant long-term surveillance is critical, as delayed or atypical presentations may otherwise be missed. Biopsy of newly detected nodules is recommended to distinguish between inflammatory changes and viable tumor, given their radiologic overlap [11,12]. From a clinical perspective, these findings underscore the importance of careful procedural planning when performing percutaneous ablation for RCC. Technical considerations such as minimizing probe manipulation, limiting multiple needle passes, judicious use of intraprocedural biopsy, and consideration of tract ablation may help reduce the risk of tumor seeding, although supporting evidence remains limited. In addition, structured and long-term imaging surveillance, including assessment of the probe entry site, should be considered, particularly in patients undergoing repeated ablation procedures or those with higher-risk tumor features. Due to the limited number of cases and heterogeneity of reporting, no statistical correlation could be established. In addition, incomplete and inconsistent documentation of procedural details and surveillance strategies across studies restricts meaningful comparison and precludes robust inference regarding risk factors or preventive measures. These findings are consistent with broader trends in salvage RCC literature, where small sample sizes, retrospective designs, and inconsistent reporting of complications hinder evidence synthesis [10,15,16]. Nevertheless, descriptive patterns suggest possible associations with percutaneous biopsies, multiple needle passes, and repeated ablation cycles. However, given the small number of reported cases and the heterogeneity of procedural reporting, no definitive conclusions regarding causality can be drawn. These observations should therefore be interpreted as associations derived from limited observational data.

Given the rarity of tumor seeding and the limitations of existing evidence, including small sample sizes, retrospective designs, and inconsistent reporting, collaborative multicenter registries and prospective studies are essential to accurately define incidence, identify procedural and biological risk factors, and refine surveillance protocols.

## 6. Conclusions

Needle tract tumor seeding following ablative therapies for RCC remains an exceptionally rare complication. While percutaneous ablation is an effective and minimally invasive alternative to surgery, available evidence suggests that careful attention to procedural factors, such as tract ablation, minimizing multiple probe passes, and judicious use of intra-procedural biopsy, may help reduce the risk of tumor seeding. Long-term surveillance, including assessment of the probe entry site, should be considered, particularly for patients undergoing repeated treatments. With appropriate patient selection, thorough counseling, and meticulous technique, ablation remains a safe and valuable treatment option for SRMs. However, current preventive and surveillance recommendations are based primarily on limited observational data. Future prospective and standardized studies are needed to better define the true incidence of tumor seeding, clarify procedural and biological risk factors, and refine preventive strategies.

## Figures and Tables

**Table 1:** Study and Patient Characteristics.

Author	Year	Design	Cases Number	Modality	Age (Year)	Patient Gender
Krambeck [9]	2005	CR	1	RFA	66	Woman
Akhavein [7]	2012	CR	1	CA	84	Man
van de Kamp [10]	2015	CR	1	CA	65	Woman
Viswanathan [11]	2015	CS	2	CA	63	Woman
Rizzo [12]	2019	CS	2	CA	72	Man
Shen [13]	2019	CR	1	CA	69	Man
Fichtenbaum [14]	2019	CR	1	CA	76	N/A
Rizzo [15]	2020	CR	2	CA	47 and 66	Men

Abbreviations: CR, Case Report; CS, Case Series; RFA, Radiofrequency Ablation; CA, Cryoablation; N/A, Not Available.

**Table 2:** Tumor and Needle Manipulation Characteristics.

Initial Procedure	Histology (Subtype)	Fuhrman Grade	Needle Tumor Manipulation	Needle (Type)	Probe (Number and Type)	Tract Ablation
Open NSS	ccRCC	2–4	Three separate RFA cycle	N/A	N/A	Yes
PC biopsy	ccRCC	N/A	CA Biopsy	N/A	N/A	N/A
LRP biopsy	ccRCC	1	Biopsy, and two 10 min LRP CA cycles	16-gauge	Four IceSeed	N/A
CT-guided PC biopsy	ccRCC	2	CA Biopsy CA	19-gauge coaxial 20-gauge	Four Perc-24	N/A
Open NSS	pRCC	N/A	Biopsy Two CA cycles Biospy	20-gauge	N/A	N/A
CT-guided PC biopsy	RCC	Not reported	Biopsy Two CA cycles Biospy	20-gauge coaxial	Three cryoprobes N/A	N/A
PC biopsy	ccRCC	3	Biopsy Two separate CA	N/A	N/A	N/A
Open NSS	RCC	1	Two 10 min CT-guided CA cycles Biopsy	N/A	Two 17-gauge	N/A
NSS	pRCC	N/A	Two 10 min CA cycles Three repeat CA Biopsy Two 4.5 min CA cycles, and Two 3 min cycles	N/A	Two 17-gauge Two 10-gauge	N/A

Abbreviations: CA, Cryoablation; ccRCC, clear-cell Renal Cell Carcinoma; CT, Computed Tomography; LRP, Laparoscopic Retroperitoneal; N/A, Not Available; NSS, Nephron-Sparing Surgery; PC, Percutaneous; pRCC, papillary Renal Cell Carcinoma; RCC, Renal Cell Carcinoma; RFA, Radiofrequency Ablation.

**Table 3:** Follow-up and Outcomes of Tumor Seeding.

Follow-Up and Timing (Months)	Time to Seeding (Months)	Clinical Presentation	Site	Treatment	Histology (Subtype)	Clinical Outcome
CT scan 3, and 6	9	Three 1 cm masses, and one 4 cm mass	Paracolic gutter, and cul-de-sac of Douglas	Exploratory laparotomy, and resection of the metastases	ccRCC Fuhrman 3–4	N/A
CT scan 3	3	Numerous nodules	Retroperitoneal, and subcutaneous fat	Palliative care	N/A	Progression to metastatic disease; Alive at 12 months
CT scan 60	60	Several 3–7 mm nodules	Perinephric fat	Laparoscopic RN	ccRCC	No recurrence at 24 months
CT scan 4, and 10	10	One 4 cm lobulated mass	Flank musculature	Locally excision	ccRCC Fuhrman 2	Progression to metastatic disease; Exitus at 24 months
CEUS and MRI 3	3	Three solid lesions	Perinephric fat, and lumbar muscle	CA	pRCC	N/A
CT scan 3, 9 and 21	21	Two lesions of 2.7, and 3.8 cm	Ablation site, rectovesical pouch, right ureter, right seminal vesicle, and rectum	Salvage cytoreductive RARN, resection of pelvic mass, ureteral stent, and systemic therapy	ccRCC sarcomatoid Fuhrman 4	No recurrence at 10 months
CT scan 12, and 16	16	N/A	Cryoprobe tracts	N/A	N/A	N/A
MRI 6, and 12	12	Abdominal mass	Posterior flank musculature	Chemotherapy	Undifferentiated RCC	Progression of the disease; Exitus at 6 months
CEUS and CT scan N/A	N/A	One 2.7 × 1.6 cm solid lesion, and one smaller lesion	Posterior perinephric fat, and perinephric fat	CA	pRCC	No recurrence at 12 months

Abbreviations: CA, Cryoablation; ccRCC, clear-cell Renal Cell Carcinoma; CEUS, Contrast-Enhanced Ultrasound; CT, Computed Tomography; MRI, Magnetic Resonance Imaging; N/A, Not Available; pRCC, papillary Renal Cell Carcinoma; RARN, Robotic-Assisted Radical Nephrectomy; RCC, Renal Cell Carcinoma; RN, Radical Nephrectomy.

## Data Availability

The datasets generated during and/or analyzed during the current study are available from the corresponding author on reasonable request.

## Abbreviations

The following abbreviations are used in this manuscript:

CA	Cryoablation
ccRCC	clear-cell Renal Cell Carcinoma
CEUS	Contrast-Enhanced Ultrasound
CR	Case Report
CS	Case Series
CT	Computed Tomography
IQR	Interquartile Range
MRI	Magnetic Resonance Imaging
MWA	Microwave Ablation
NSS	Nephron-Sparing Surgery
pRCC	papillary Renal Cell Carcinoma
PRISMA	Preferred Reporting Items for Systematic Reviews and Meta-Analyses
RCC	Renal Cell Carcinoma
RFA	Radiofrequency Ablation
SRMs	Small Renal Masses
